# Implementing Oncology Pharmacy Boot Camp for Advanced Pharmacy Practice Experience Students at a Regional Conference in Saudi Arabia

**DOI:** 10.7759/cureus.34780

**Published:** 2023-02-08

**Authors:** Mohammed Alnuhait, Atika Alharbi, Abdalrhman Alanizi, Nora Alkhudair, Majed Alshamrani, Eshtyag Bajnaid, Abdullah Alrajhi, Fouad Alnajjar, Amr Khardaly

**Affiliations:** 1 Department of Clinical Pharmacy, Umm Al-Qura University, Makkah, SAU; 2 Department of Pharmaceutical Care Services, King Abdulaziz Medical City (KAMC), Jeddah, SAU; 3 Department of Pharmaceutical Care, King Abdullah bin Abdulaziz University Hospital, Riyadh, SAU; 4 Department of Clinical Pharmacy, College of Pharmacy, King Saud University, Riyadh, SAU; 5 Department of Pharmaceutical Care Services, King Abdullah Medical City, Makkah, SAU; 6 Department of Clinical Pharmacy, King Fahad Medical City, Riyadh, SAU; 7 College of Pharmacy, Alfaisal University, Riyadh, SAU; 8 Deputyship of Therapeutic Affairs, Ministry of Health, Riyadh, SAU

**Keywords:** sopa, advanced pharmacy practice experience, pharmacy education, oncology, boot camp

## Abstract

Background

Upon graduation, pharmacy students are expected to possess a diverse array of knowledge, skills, and attitudes. A subject-specific boot camp may support bridging the gap between the information and skills gained during clerkships, courses required for pharmacy school after graduation, and skills needed for the job market, as well as the gap between pharmacy school and residency. This research aimed to determine whether an integrated boot camp increased Advanced Pharmacy Practice Experience (APPE) student self-confidence and enhanced students’ knowledge in oncology pharmacy.

Method

APPE students who attended the annual meeting of the Saudi Oncology Pharmacy Assembly (SOPA)/International Society of Oncology Pharmacy Practitioners (ISOPP) Regional Conference 2021 were voluntarily enrolled in a three-hour oncology-focused boot camp consisting of interactive lectures. Pre- and post-intervention examinations were used to evaluate student learning outcomes and their experience feedback.

Result

Of 118 students who attended the boot camp, 80 students who met the criteria were included in the study. The pre- and post-intervention examinations were completed by the 80 APPE students. The pre-intervention test results (mean: 66%, standard deviation (SD): 16%) increased by 21.5% after the boot camp (mean: 87.5%, SD: 12%, p = 0.001). Students scored better on each of the 10 test questions, with nine questions demonstrating a statistically significant result.

Conclusion

The results of this research showed that interns who participated in an oncology boot camp had a higher level of knowledge and confidence in applying key oncology concepts. Interns were satisfied with the chance to engage in the activity, and they agreed to adding boot camps to future conferences would be valuable. This research shows that it is possible to hold a transitional boot camp during conferences to better prepare students for their fields of study and increase their participation in oncology conferences.

## Introduction

Upon graduation, pharmacy students are expected to possess a diverse array of knowledge, skills, and attitudes [1]. The Saudi Commission for Health Specialties (SCFHS) has adopted laws to review and standardize the credentials of physicians, nurses, dentists, and pharmacists [1]. SCFHS classifies health certificate holders, evaluates their credentials, establishes the foundations and requirements for practicing health professions, and licenses health practitioners professionally [2]. The role of SCFHS is to ensure that the healthcare professional has the essential skills, knowledge, and competencies to diagnose and treat the condition optimally and safely. In addition, they are involved with the training of healthcare professionals by overseeing the scientific and professional training programs included in the Saudi Specialty Certificate and Diploma. SCFHS provides Saudi licensure examination to identify the competencies required to earn a license to practice the profession after the internship year, which, in turn, ensures that graduates of approved health education institutions satisfy the basic requirements for practice [2].

The Saudi Pharmacist Licensure Examination (SPLE) was developed to evaluate the competency of pharmacy school graduates to assure pharmacists’ competence [1]. Approximately 35% of the assessment is composed of clinical sciences, which is one of the examination’s sections that include oncology as a core module [1].

Pharmacy schools across the country have varying curriculums that aim to prepare students to successfully pass the examination and subsequently enter the job market. In a local single-institution study, a correlation was shown between achieving good marks in therapeutic courses and achieving a higher SPLE score [3]. Several studies have looked at evaluating additional extracurricular activities to complement knowledge levels. Advanced preparedness courses may assist in bridging the gap between the knowledge and abilities acquired during clerkships and courses that meet the expectations for medical school after graduation and bridge the gap to residency [4]. A boot camp consisting of extensive cognitive and skill training may assist students in practicing clinical skills [5]. Furthermore, residency programs have implemented boot camps to impart clinical skills to their residents and shown positive results [6,7]. Despite the growing number of pharmacy graduates who obtain a license to practice the profession after the internship year, there are a limited number of specialized pharmacists in Saudi Arabia [8].

Oncology pharmacists are essential members of the oncology multidisciplinary teams, responsible for comprehensive medication management, patient counseling, therapeutic plan development, formulary management, drug therapy evaluation, safe compounding and preparation of chemotherapy, and treatment-related toxicity monitoring [9]. Based on the update to the American College of Clinical Pharmacy (ACCP) pharmacotherapy didactic curriculum toolkit, oncological conditions are classified as tier 2, which means that the students should receive education and training on this topic, with additional knowledge or skills may be required after graduation such as residency training or equivalent experience [10]. In our experience, students have limited exposure to cancer-related drugs, diseases, and associated complications during Advanced Pharmacy Practice Experience (APPE). As a result of this, students may require a significant amount of time during their rotations to relearn basic oncology.

To our knowledge, this is the first study to evaluate the effectiveness of a boot camp and its role in motivating students and advancing their knowledge in the oncology field combined with attending a regional conference. The purpose of this study was to assess whether an integrated boot camp raised student self-confidence and improved their knowledge and abilities in oncology pharmacy. The regional conference was organized by the Saudi Oncology Pharmacy Assembly (SOPA). SOPA was established in late 2018 as a subdivision of the Saudi Oncology Society (SOS) to promote the professional services offered by oncology pharmacy practitioners in Saudi Arabia and the Gulf region. SOPA held its annual regional hybrid conference in Riyadh, Saudi Arabia, in partnership with the International Society of Oncology Pharmacy Practitioners (ISOPP). The scientific program was presented over three days, from November 18th to November 20th, 2021 [11].

## Materials and methods

All fourth-year APPE students (P4) who attended the conference physically or virtually were included in the study; however, those who did not complete the survey were excluded from the study and analysis. Modules were included in the boot camp as shown in Table 1: Introduction to Oncology and Scope of Oncology Pharmacy Practice, Review of Chemotherapy, Review of Chemotherapy Toxicities, Review of Immunotherapy, and Review of Targeted Agents. The day before the boot camp, investigators provided APPE students with comprehensive instructions for the boot camp. The participants then completed a pre-intervention test comprising 10 knowledge/skills questions and demographic data. In addition, they were given a pre-intervention survey to assess their perceptions of their oncology pharmacy knowledge and their satisfaction with the program.

During the conference, two oncology-specialized pharmacists led a three-hour interactive session on topics that every oncology pharmacist should be familiar with. After the session concluded, the students retook both the test and the questionnaire, which included questions about course satisfaction. Throughout the boot camp, the Mentimeter program (an interactive presentation software using real-time voting) (Stockholm, Sweden) was utilized. There was also a competition for all attendees following the sessions. Approximately 118 students participated, and the top five scorers in the competition were recognized and awarded prizes. The Institutional Review Board of Umm Al-Qura University approved this research. The researchers obtained informed consent from the participants.

**Table 1:** Boot camp program

Boot camp program
Introduction to Oncology and Scope of Oncology Pharmacy Practice
Review of Chemotherapy (all classes of chemotherapies)
Review of Chemotherapy Toxicities (most common toxicities in each class)
Review of Immunotherapy (class of immunotherapies and their role in oncology and management of side effects)
Review of Targeted Agents (targeted agents used commonly in oncology and management of side effects)

Data were reported as means and standard deviations (SDs), medians, and ranges for continuous variables. To describe categorical variables, proportions were used. Using chi-square tests, categorical data were evaluated. T-tests were used to evaluate continuous variables whose distributions were near normality. Statistical significance is defined as p < 0.05. All statistical analyses were performed using the Statistical Package for the Social Sciences (SPSS) version 21.0 (IBM SPSS Statistics, Armonk, NY, USA).

## Results

Table 2 shows the demographic characteristics of the APPE students who participated in the boot camp. The median age of the participants in this study was 23 years, 76% were female, and 86% of the participants were Saudi citizens. Hail University had the highest percentage of participants in the boot camp, with 16% of all participants, followed by King Saud University, with 14% of all participants, and the remaining represented various universities from the Gulf region. Nearly 39% of the participants attended the boot camp in person, while 61% participated virtually. Of the group, 85% had studied oncology pharmacotherapy in pharmacy school. Pre-boot camp knowledge of oncology pharmacotherapy was rated as average by 47% of the attendees and poor by 21%. Of those who attended the boot camp, 70% had never taken oncology clinical rotations before. The pre- and post-intervention tests were completed by 80 APPE students. Of 118 (68%) students who attended the boot camp, 80 met the criteria and were enrolled in the research.

**Table 2:** Demographic characteristics of APPE students enrolled in the oncology pharmacy boot camp Others: Imam Abdulrahman Bin Faisal University, King Faisal University, North Border University, Batterjee Medical College, and Taif University IQR: interquartile range

Characteristics	Value
Age (years)	Median (IQR)
	23 (21-25)
Gender	% (number)
Female	76.25% (61)
Male	23.75% (19)
Nationality	
Saudi	86.25% (69)
Non-Saudi	13.75% (11)
College	
Hail University	16.25% (13)
King Saud University	13.75% (11)
Umm Al-Qura University	12.5% (10)
Ibn Sina National College for Medical Studies	8.75% (7)
King Saud bin Abdulaziz University for Health Sciences	7.5% (6)
Princess Nourah Bint Abdulrahman University	6.25% (5)
Almaarefa University	6.25% (5)
King Abdulaziz University	5% (4)
Jazan University	3.75% (3)
Prince Sattam bin Abdulaziz University	3.75% (3)
Qassim University	3.75% (3)
Qatar University	3.75% (3)
Taibah University	2.5% (2)
Others*	6.25% (5)
Attending boot camp	
Virtually	61.25% (49)
Physically	38.75% (31)
Previous oncology pharmacotherapy course	
Yes	85% (68)
No	15% (12)
How do you assess your knowledge of oncology pharmacy before attending this session?	
Extremely excellent	8.75% (7)
Good	22.5% (18)
Average	47.5% (38)
Poor	17.5% (14)
Very poor	3.75% (3)
Have you received clinical training (rotation) in oncology?	
Yes	30% (24)
No	70% (56)

Table 3 demonstrates that pre-intervention test results (mean: 66%, SD: 16%) increased by 21.5% after the boot camp (mean: 87.5%, SD: 12%, p = 0.001). Students scored better on each of the 10 test questions, with nine questions demonstrating a statistically significant result. Table 4 and Table 5 provide the survey questions about pre- and post-intervention perceptions.

**Table 3:** Students’ test performance on the knowledge-based evaluation The level of statistical significance is set at p < 0.05. A paired t-test was used.

Assessment questions	Average pre-intervention score (N = 80 students) (% (number))	Average post-intervention score (N = 80 students) (% (number))	P-value*
1. Oncology pharmacy practice	82 (66)	85 (68)	0.2078
2. Doxorubicin toxicity management (extravasation)	62.5 (50)	88.75 (71)	<0.001
3. Chemotherapy toxicities	72.5 (58)	90 (72)	<0.001
4. Chemotherapy-induced nausea and vomiting management	60 (48)	92.5 (74)	<0.001
5. Methotrexate supportive care	68.5 (55)	95 (76)	<0.001
6. Identifying immune checkpoint inhibitors	58.8 (47)	80 (64)	<0.001
7. HER2-positive breast cancer treatment	80 (64)	88.75 (71)	0.036
8. Carboplatin dose calculation	66.3 (53)	90 (72)	<0.001
9. Palliative care and hospice care	63.7 (51)	80 (64)	<0.001
10. Chemotherapy-induced diarrhea management	45 (36)	85 (68)	<0.001
Overall score	66%	87.5%	<0.001

**Table 4:** Students’ perceptions of their knowledge in oncology 1 = very poor, 2 = poor, 3 = average, 4 = good, 5 = extremely excellent The level of statistical significance is set at p < 0.05. A paired t-test was used.

Survey question	Pre-boot camp (% (number))	Post-boot camp	P-value*
How do you assess your knowledge in oncology pharmacotherapy? (students graded themselves as good and excellent)	31.2 (25)	85 (68)	<0.001

**Table 5:** Students’ responses to a satisfaction survey following the oncology pharmacy boot camp

Survey questions	Strongly agree (% (number))	Somewhat agree (% (number))	Neutral (% (number))	Somewhat disagree (% (number))	Strongly disagree (% (number))
1. The oncology pharmacy boot camp aided in the comprehension of oncology topics.	90% (72)	10% (8)	0% (0)	0% (0)	0% (0)
2. The event’s duration was just right (neither too long nor too brief).	27.5% (22)	41.25% (33)	18.75% (15)	5% (4)	7.5% (6)
3. The event met my expectations.	93.75% (75)	6.25% (5)	0% (0)	0% (0)	0% (0)
4. I am satisfied with the event overall.	86.25% (69)	1.25% (1)	8.75% (7)	0% (0)	3.75% (3)
5. The event was interactive.	65% (52)	0% (0)	0% (0)	35% (28)	0% (0)
6. The event helped me acquire new insights and knowledge.	98.75% (79)	0% (0)	0% (0)	0% (0)	1.25% (1)
7. The speakers or presenters were knowledgeable.	98.75% (79)	0% (0)	0% (0)	0% (0)	1.25% (1)
8. The event has been well-planned and organized.	75% (60)	12.5% (10)	12.5% (10)	0% (0)	0% (0)
9. In the future, I will likely attend one of SOPA’s events.	98.75% (79)	0% (0)	0% (0)	0% (0)	1.25% (1)

Students’ test scores before and after the oncology pharmacy boot camp were compared on knowledge-based evaluation questions, and the results showed a statistically significant improvement in all domains except the scope of the oncology pharmacy practice domain. By the end of the oncology pharmacy boot camp, 85% of the students had rated themselves as having good or excellent oncology knowledge, which is a significant increase from their pre-boot camp ratings. The results of a satisfaction survey given to students after the oncology pharmacy boot camp revealed that the students had a positive experience and that the boot camp had helped them better understand oncology-related concepts.

## Discussion

In the past few years, there has been a rise in the popularity of the “boot camp” as a popular method for education and learning [6]. This might be related to a greater awareness of the limits of conventional training methods and the development of novel techniques to encourage active learning and enhance foundational expertise [12,13]. Active learning focuses on higher-order Bloom’s taxonomy levels, such as application, analysis, synthesis, and assessment, and is thought to improve students’ long-term recall of material [12].

Our study aims to evaluate the effectiveness of an oncology boot camp and its role in motivating students and advancing their knowledge in the oncology field combined with attending regional conferences. The primary goal of this boot camp session was to build a self-contained learning resource that would help students get a better grasp of fundamental oncology principles. We went with several sessions during the boot camp. To achieve this goal, we made use of interactive technologies, such as an interactive platform (Mentimeter).

Since pharmacy students are expected to get exposed to oncology either through clerkships or during didactic courses in pharmacy school, our boot camp may have been considered a refresher course. Nonetheless, we sought to determine if their confidence in executing oncology crucial skills would increase after completing this refresher boot camp. The oncology pharmacy boot camp proved to be an effective instructional tool that improved student knowledge and self-assurance in navigating basic oncology pharmacy knowledge [14].

Confidence is a component of the crucial notion of self-efficacy on a broad level. In the context of medical education, it has been shown that confidence is essential for trainees’ capacity to lead and acquire team trust, and that confidence correlates strongly with trainees’ competency [15,16]. Our main aim was to encourage students, particularly those who are at the internship level, to attend our conference, and we provided them with relevant content within the conference agenda, thus further fostering in them a spirit of enthusiasm and a desire to network with experts at a scientific conference.

The conference involved two masterclasses, as well as students’ boot camp and competition, with over 100 students in attendance. The scientific program was delivered by 34 international speakers and moderators, who discussed recent developments in oncology pharmacy treatments, practice management, and cancer research. The SOPA/ISOPP 2021 regional conference was a success story, with 1,000 practitioners participating both virtually and in person. This one-of-a-kind conference was held to improve the knowledge and skills of oncology pharmacy practitioners in Saudi Arabia and the region, which will reflect on the quality of pharmaceutical care provided to cancer patients following the 2030 vision for improving the healthcare system and quality of life [17].

To our knowledge, this is the first study to evaluate the impact of holding a boot camp on the side of a regional conference and its role in motivating students and advancing their knowledge in the oncology field. Students gave the boot camp a high rating and were satisfied with this experience. It did not surprise the researchers that the average pre-intervention test score was about 66%. The questions focused on issues that are routine in the field of oncology. For example, an intern who is doing an APPE rotation in oncology will need to know how to use the Calvert formula to determine the appropriate dosage of carboplatin, yet this is not an equation or method that students are likely to have mastered in advance. As the numbers show, this is the case: before attending the boot camp, only 66% of students could correctly remember and apply the Calvert formula, but after attending the boot camp, over 90% successfully applied the equation. The overall rise in post-assessment is almost 21%. All questions proved that the student’s knowledge in previously weak areas had significantly increased attributable to the boot camp.

Moreover, to measure the effectiveness of the cancer boot camp, a survey of student participants was developed and distributed. After the boot camp, students reported greater ease with the oncology-related topics surveyed. An interesting research was reported in the area of oncology pharmacy, whereby they demonstrated the efficacy of a virtual session for the delivery of a boot camp setting [14].

Our research also supports the findings by Serag-Bolos et al. that non-traditional ways of delivering information enhance trainees’ knowledge, skills, and confidence [18]. The boot camp was a successful learning experience that helped students gain a deeper understanding of fundamental oncology principles and build confidence in their ability to use that information in real-world situations [14].

Several limitations hinder this study because of its limited focus. The first limitation is the small sample size, which makes it difficult to generalize the findings to all APPE pharmacy students. However, they were a diverse group of students attending different universities. In addition, there was no comparison group; thus, it was difficult to ascertain whether the same gains in knowledge and self-confidence would have been made via more conventional means of education. Another limitation concerns the study’s follow-up and the boot camp’s long-term effectiveness. The faculty had to put significant time and effort into designing, producing, and managing the oncology boot camp experience. Based on our results, we recommend adding boot camps or advanced learning experiences to pharmacy conferences to augment the engagement of pharmacy interns and students and enhance their knowledge of the different aspects of pharmacy practice and increase their participation in pharmacy conferences.

## Conclusions

The results of this research showed that interns who participated in an oncology boot camp had a higher level of knowledge and confidence in applying key oncology concepts. Interns were satisfied with the chance to engage in the activity, and they agreed to include boot camp in future conferences. This research shows that it is possible to hold a transitional boot camp during conferences to better prepare students for their fields of study and increase their participation in oncology conferences.
